# *In silico* Discovery of Novel FXa Inhibitors by Pharmacophore Modeling and Molecular Docking

**DOI:** 10.1007/s13659-017-0126-x

**Published:** 2017-06-02

**Authors:** Yinglan Pu, Hui Liu, Yeheng Zhou, Jiale Peng, Yaping Li, Penghua Li, Yingying Li, Xingyong Liu, Li Zhang

**Affiliations:** School of Chemical Engineering, Sichuan University of Science & Engineering, Zigong, China

**Keywords:** FXa, Thrombotic diseases, Pharmacophore, Docking

## Abstract

**Abstract:**

Coagulation Factor Xa (FXa) is the crucial enzyme at the convergent point of the intrinsic and extrinsic coagulation pathways. The inhibition of FXa is an effective approach against thrombotic diseases. In the present study, a specific strategy is reported to discover 10 novel FXa inhibitors based on ligand-based (pharmacophore) virtual screening and molecular docking analysis from a dataset of specs(containing 220000 molecules). The binding modes analysis provide insights into the contribution of particular structural moieties of the compounds towards their activity against FXa, and 10 novel structural compounds were discovered as potent candidate molecules. This work could be helpful in further design and development of FXa inhibitors.

**Graphical Abstract:**

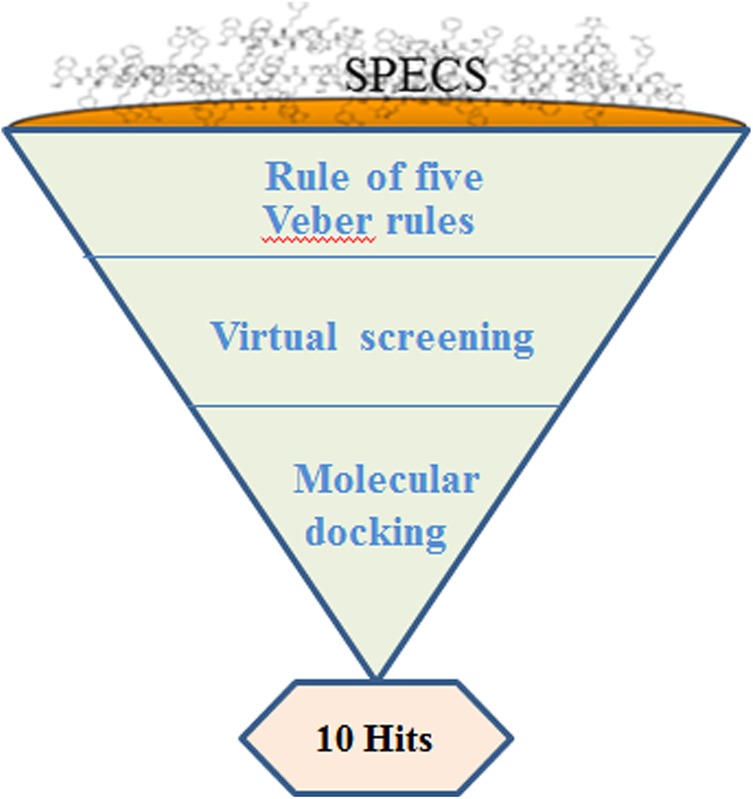

## Introduction

FXa, a vitamin K-dependent serine protease, is a key enzyme in the coagulation cascade [1]. It is located at the convergence of the extrinsic and the intrinsic activation pathways [2]. Due to it is upstream of thrombin, FXa can promote the production of new thrombin without causing bleeding symptoms or affecting the existing thrombin [3]. Therefore, FXa is recognized as an ideal and attractive target for anticoagulation therapy in the development of new drugs, which make FXa inhibitors become a hot topic of anticoagulant research [4].

Thrombotic diseases, such as myocardial infarction and stroke, could cause organ tissue ischemia, necrosis, seriously endanger human health [5, 6]. Anticoagulants are widely used in the prevention and treatment of thromboembolic disorders. Pre-existing anticoagulant drugs, such as unfractionated heparin, low molecular weight heparin, warfarin, have some limitations: narrow treatment window, slow onset, causing bleeding [7–12]. Owing to above reasons, these drugs have been gradually replaced by anticoagulant drugs targeting a single coagulation factor [13].

**Fig. 1:**
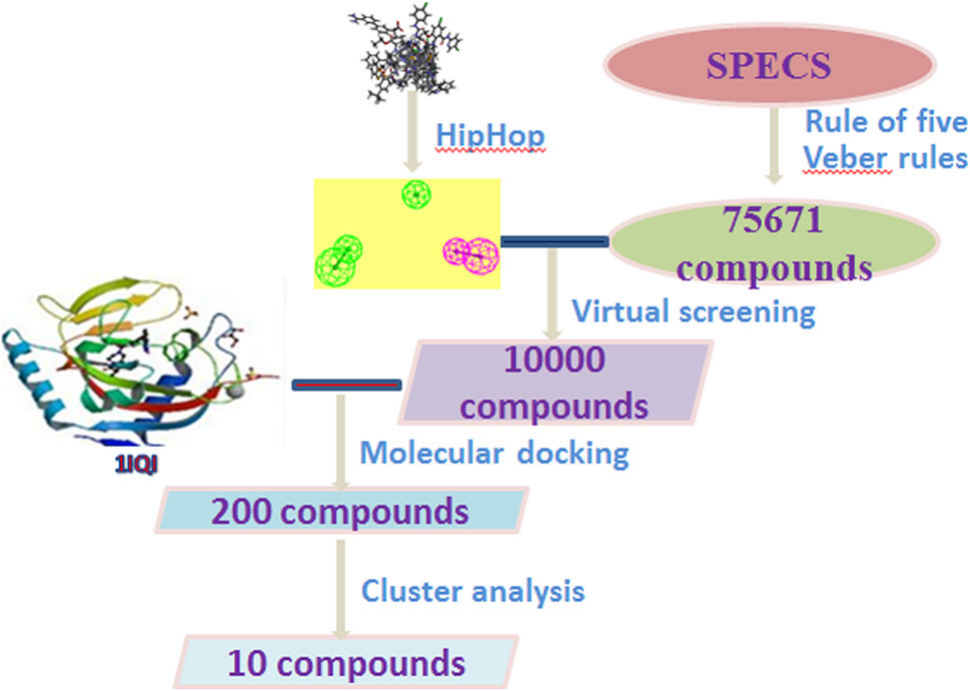
Overview of the workflow

In order to develop novel FXa inhibitors, we have developed a comprehensive and rational protocol. Herein, a series of new hits were discovered based on ligand-based screening and molecular docking (Fig. 1). The specs database was first refined according to the Rule of five and Veber rules. Secondly, the pharmacophore features based on the known inhibitors were employed to query the new potent inhibitors. Thirdly, docking strategy was used to screen the remained top 10000 compounds. Eventually, 10 potential FXa inhibitors, which were obtained according to the docking score list, were further studied on their interactions with FXa.

## Materials and Methods

Virtual screening is one of the most widely applied methods to discover novel scaffolds for various targets. Generally, it can be categorized into two major types: structure-based method and ligand-based method [14]. In the present research, to optimize the development of new FXa inhibitors, docking strategy and ligand-based pharmacophore were used in combination to identify the novel potential FXa inhibitors.

The GOLD 5.2.2 (Genetic Optimization of Ligand Docking) [15], incorporated installed on Intel(R) Xeon(R) CPU 2.30 GHz, 32 CPU, Core 8 Dell server with Linux operating systems, was used for molecular docking studies. While pharmacophore was carried out using pharmacophore module from Discovery Studio Version 4.0 (DS 4.0, Accelrys Inc., San Diego, CA), which is commercially available software and widely used in the drug discovery. The specs database consists of about 220000 small molecules, downloaded from specs official website (www.specs.net). Figure 1, including molecular docking and ligand-based (HipHop pharmacophore) screening, showed the working flow chart of this study.

### Dataset

**Fig. 2:**
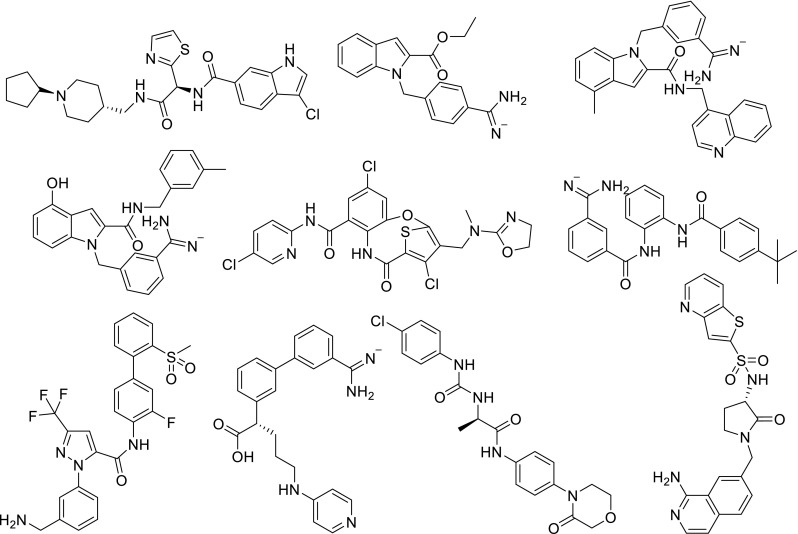
Chemical structures of the training set compounds

A dataset of 24 FXa inhibitors were collected from different publications, 10 of which were selected as training set to generate common features (Fig. 2). The other 14 molecules were treated as active compounds of test set. While the 14 inactives were randomly chosen from FXa-decoys set reported by University of California, San Francisco (http://dud.docking.org). For each compound, the geometries were corrected, atoms were typed and energy mini-mization was performed using DS 4.0.

The X-ray crystal structure of FXa complex with an inhibitor M55125 (PDB ID:1IQI) [16] with high resolution of 2.9 Ǻ was downloaded from the RCSB Protein Data Bank (www.rcsb.org). Before docking, the obtained protein was prepared in the Accelrys DS 4.0. The water was removed, some other co-crystallized small molecules were deleted, hydrogens were added, and the whole structure was optimized using SYBYL 2.0.

### Pharmacophore Generation and Validation

The HipHop pharmacophore model was developed using Pharmacophore module of DS 4.0. The training set molecules were considered to generate conformation. The parameters were set as follows. First, the Principal and MaxOmitFeat parameters were set as 2 and 0, respectively. Then, on the feature mapping procedure step, all the features were chosen, the Minimum/Maximum Features parameter was set as 0 and 5. In the final step, according to the details of results of the previous step, 6 features were selected to generate the common feature pharmacophore which were HB-ACCEPTOR, HB_ACCEPTOR_lipid, RING_AROMATIC, HBA_HEAVY, HBA_PROJECTION, and HBD_1. HB-ACCEPTOR represents Hydrogen-Bonding Acceptor, HB_ACCEPTOR_lipid represents lipidic hydrogen bond acceptor, RING_AROMATIC represents ring aromatic, HBA_HEAVY represents the heavy atom of a hydrogen bond acceptor, HBA_PROJECTION represents the projection point of a hydrogen bond acceptor, and HBD_1 represented hydrogen bond donor and matching an aromatic carbon with a hydrogen. The rest of the parameters were set at default values.

Finally, 10 hypotheses (Hypo) were generated and validated by test set. Validation is an essential step to pick out the most suitable Hypo from the ten pharmacophore models. In this work, the developed pharmacophore hypotheses were validated by heat map, and the Hypotheses 4 was turned out to be the best model was then used to virtual screening.

### Virtual Screening Based on Pharmacophore Modeling

Hypo 4, the best pharmacophore, was selected to screen the database. The Number of Conformations was set to 200 and the Conformation Method was set to BEST. While Minimum Interfeature Distance was set to 2, Limit Hits was set to First N and Maximum Hits was set to 500. Most of other parameters were set as default. Eventually, the top 10000 (pharmacophore) molecules were chosen to the docking process.

### Molecular Docking

The molecular docking in this study was carried out utilizing the crystal structure of FXa (PDB ID: 1IQI). The active pocket was defined by a grid with the outer box dimensions of 8Å centered on the crystalized ligand. Before screening, we’ve tried a variety of scoring functions to obtain the optimal parameters. Among them, when the inner-ligand was re-docked into the binding pocket with the scoring function of Chemplp, the root mean square deviation (RMSD) of all atoms between docked pose and original conformations was minimum (0.410 Å), indicating that the parameters for docking simulation were good in reproducing the X-ray crystal structure. The Chemscore Kinase and ChemPLP were applied as template and scoring function. Each of the top 10000 (pharmacophore) molecules was docked 30 times. Other docking operations were performed with the default settings. Finally, 200 molecules with the highest docking scores were reserved for the further cluster analysis.

### Cluster Analysis

After the molecular docking, the top-ranked 200 molecules were further analyzed using a series of analysis methods, visual inspection of binding mode and the ligand fitness of the pharmacophore. Finally, 10 compounds, which have structural diversity, proper binding modes and good fitness, were considered as the fit conformation.

## Result and Discussion

### Pharmacophore Generation and Validation

**Fig. 3:**
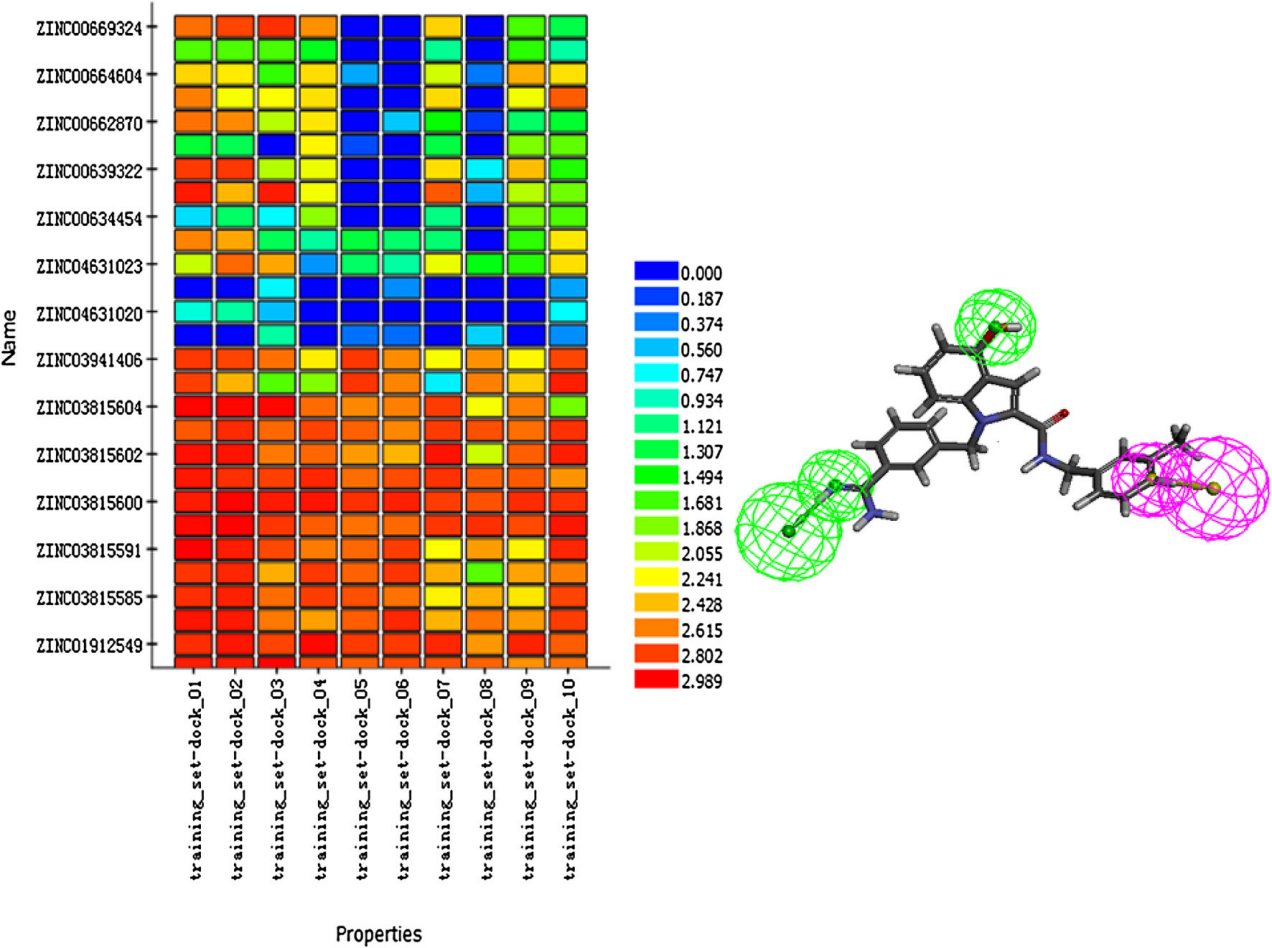
HipHop pharmacophore validation. **a**) heatmap of the ten hypotheses. **b**) the chemical features and 3D structure of Hypo 4 binding with ligand of training set. Two* colors* represented two pharmacophore features,* green* represented HBA,* magenta* represented HBD

HipHop algorithm, which is based on the common features present in the training set molecules, were applied for the pharmacophore model. Ten HipHop pharmacophore hypotheses were generated based on 10 reported FXa inhibitors of training set. The structures of the training set compounds were shown in Fig. 2. Ten hypotheses were mainly composed of two hydrogen bond acceptors (HBA), one hydrogen bond donor (HBD). Figure 3 indicated that Hypo 4 was the most desirable hypothesis. All the inactive molecules in the testing set of Hypo4, which appeared to be blue, did not fit the model well. While the active molecules could perfect satisfy well with the model. Therefore, Hypo 4 was selected as the best hypothesis to go through the next research.

### Pharmacophore Based Virtual Screening and Molecular Docking

The specs database was first screened for drug like properties using Lipinski rule of 5 and Veber rule. The remaining 75671 compounds were secondly built as a 3D database in DS 4.0. Hypo 4 was used as the model to screen the potential FXa inhibitors using DS 4.0. As a result, the 10000 compounds with top scores during the pharmacophore based virtual screening were selected for the further molecular docking.

Docking simulation of FXa was performed using the protocol of GOLD. Top 10000 compounds of scoring list of virtual screening were preserved for the molecular docking. And retain the first 200 molecules of docking to further cluster analysis.

### Clustering and Interaction Analysis

**Fig. 4:**
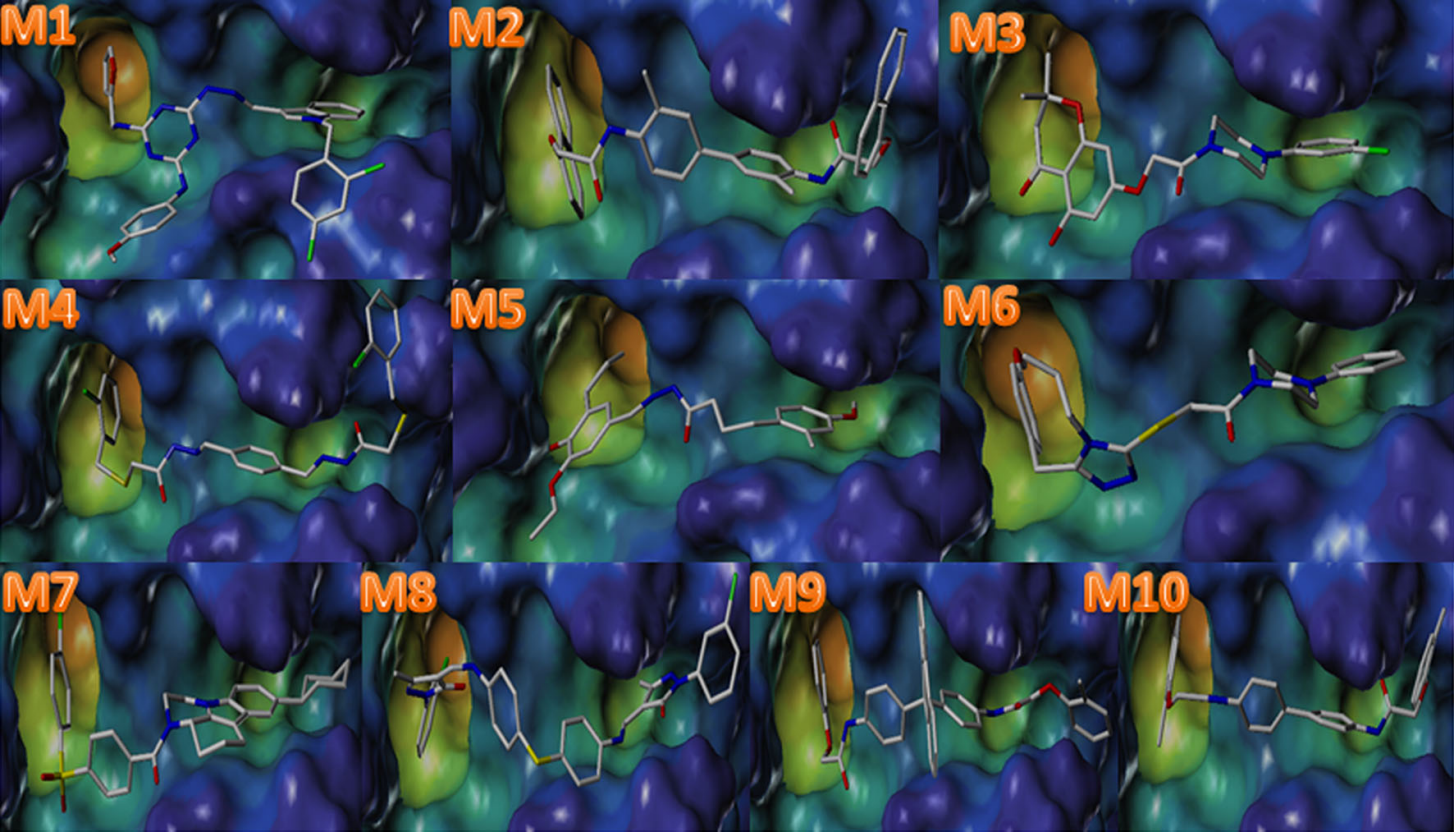
The binding mode of hits with FXa carried out by SYBYL.* Grey* sticks were represented the *ligand*, and *blue* and *green* represented active cave

**Fig. 5:**
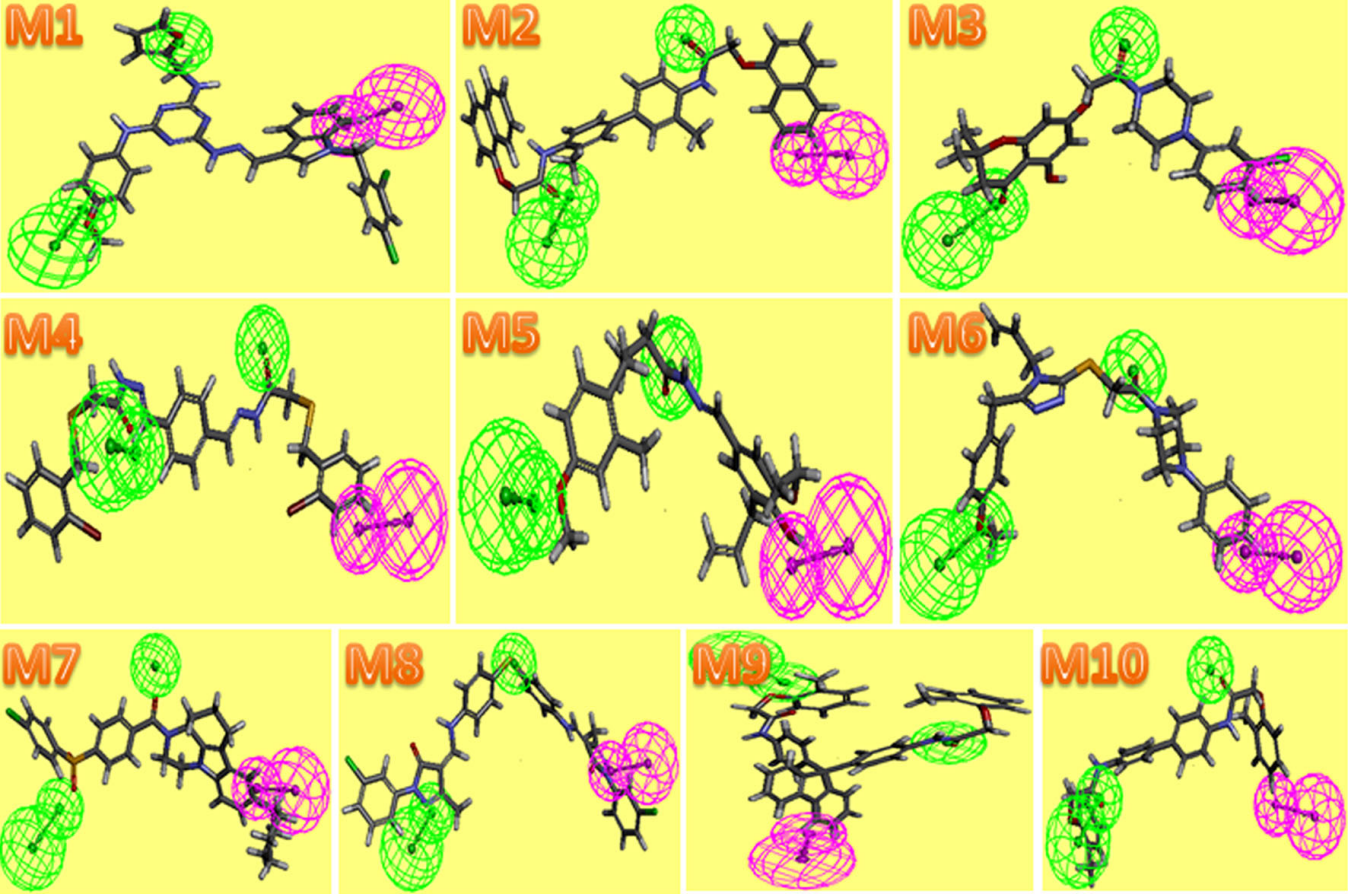
3D structure of Hypo 4 binding with hits carried out by DS. Two *colors* represented two pharmacophore features, *green* represented HBA,* magenta* represented HBD

**Fig. 6:**
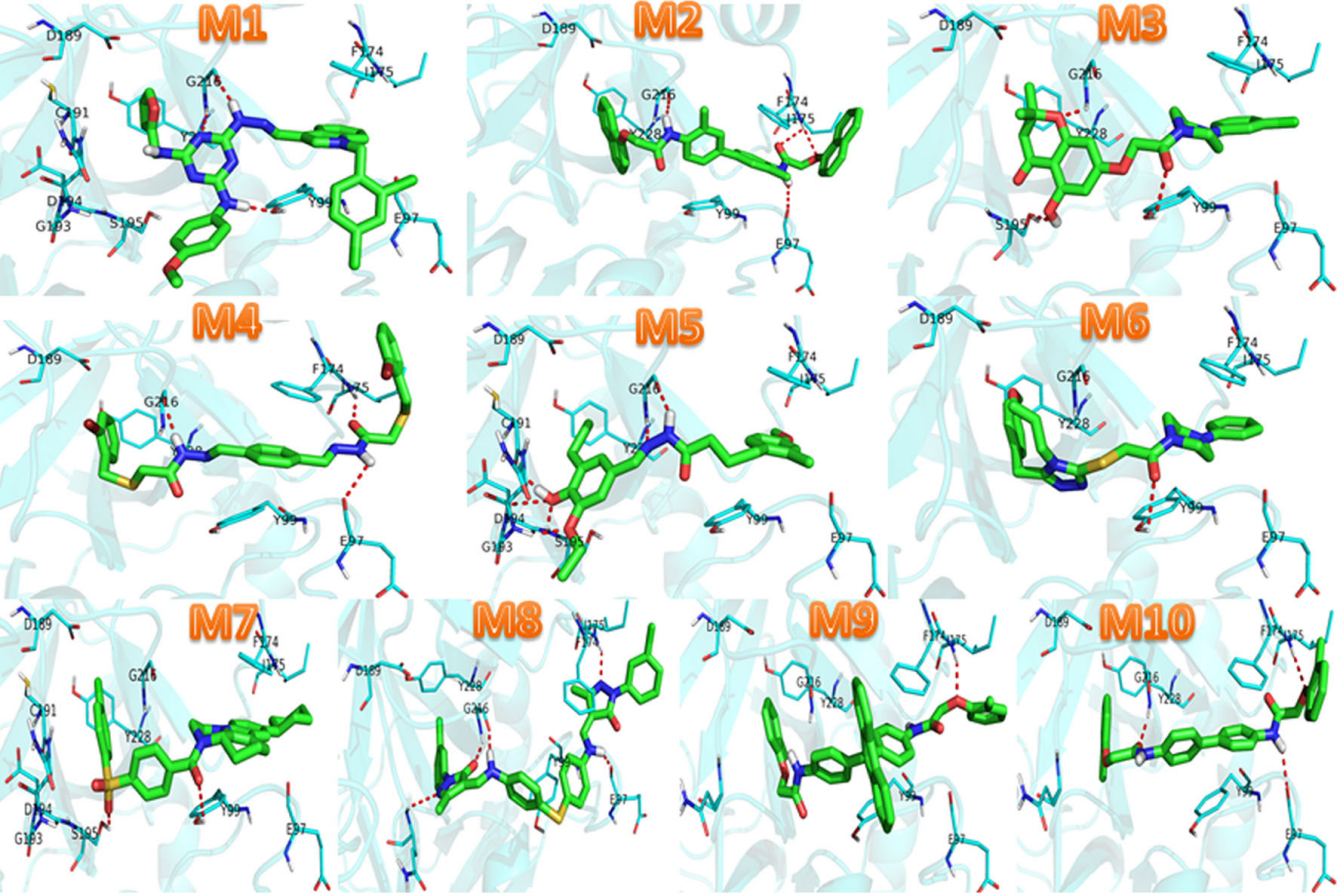
The interactions between hits and FXa carried out by Pymol.* Skyblue* sticks were represented the residues in FXa, and the ligand was *green*, *red dash line* represented hydrogen bonds

Table 1 was picked out from the top 200 of docking list by analyzing the binding modes, binding affinity and other properties based on clustering analysis, pharmacophore modeling and docking simulation. Moreover, during our survey, none of them has been reported as FXa inhibitor previously. The results of pharmacophore modeling and docking study were demonstrated in Figs. 4, 5 and 6. The molecular interaction pattern between the crystal structure of FXa and inhibitors were drawn by SYBYL, DS 4.0, Pymol.

**Table 1:** Structures of 10 compounds against FXa

No.	MW	Structure	No.	MW	Structure
M1	613.5		M2	580.68	
M3	444.91		M4	648.43	
M5	410.51		M6	463.6	
M7	573.15		M8	653.58	
M9	644.77		M10	480.56

As was shown in Fig. 4, 10 hits were perfect embedded in the active cave of FXa. The pharmacophore fitness of ten hits was demonstrated in Fig. 5, we can conclude that 10 hits had a proper fitness with the model. In **M1**, the indole ring of M1 is the main group which contributes to the hydrogen bond donor (HBD) interaction dominantly. However, 2 hydrogen bond acceptor (HBA) interactions were provided by the atom O of furan ring and methoxy. In **M2**, 2 atoms O of carbonyl figured as HBA, while HBD was offered by naphthalene. In **M3**, 2 atoms O of carbonyl and benzene ring provided 2 HBA and HBD, respectively. **M4** and **M10** were similar to **M2**. The atom O of carbonyl and methoxy were HBA(in **M5**, **M6**), hydroxyl (in **M5**) and benzene ring(in **M6**) were HBD. The atom N and O were closely related to HBA features, and cyclohexane was HBD features (in **M7**). In **M8**, the atom S and N were closely related to HBD. Similarly, **M9** got well with the model. The result of interactions 10 compounds with FXa were also investigated to testified the FXa inhibition of 10 molecules (Fig. 6). As it demonstrated, compounds were surrounded by E97, Y99, F174, I175, D189, G216, Y228. Hydrogen bonds, π–π stacking and multiple non-bonding interactions were formed between these 10 inhibitors and FXa. Among them, Y99, F97, I175, G216 were the residues involved in H-bond formation, F174, D189, Y228 were the residues involved in Pi-bond interactions and Van Der Waals interactions. These amino acids which were proved by other references were vital active residues of FXa. The interactions represented that compounds have high affinity to FXa, which also allowed compounds to develop to be a better inhibitor of FXa.

## Conclusions

In order to search effective FXa inhibitors, a rational combination strategy containing molecular docking and pharmacophore model based on the known FXa inhibitors was adopted in this study. Consequently, we discovered 10 molecules as the novel FXa inhibitors. Furthermore, the interaction modes between FXa inhibitors and 10 compounds were disclosed through clustering analysis, pharmacophore modeling and molecular docking studies. It showed that they could bind with the structure of FXa stably through some bonding interactions and non-bonding interactions. Among them, E97, Y99, F174, I175, D189, G216, Y228 were suggested to be crucial residues due to the formation of hydrogen bonds and π-π stacking with the ligands. All in all, these compounds are promising FXa inhibitors and provide a foundation for the further exploring for the treatment of thrombotic diseases.
